# Urinary biomarkers indicative of recovery from spinal cord injury: A pilot study

**DOI:** 10.1016/j.ibneur.2021.02.007

**Published:** 2021-02-18

**Authors:** Elani A. Bykowski, Jamie N. Petersson, Sean Dukelow, Chester Ho, Chantel T. Debert, Tony Montina, Gerlinde A.S. Metz

**Affiliations:** a Canadian Centre for Behavioural Neuroscience, Department of Neuroscience, University of Lethbridge, Lethbridge, Alberta, Canada; b Southern Alberta Genome Sciences Centre, University of Lethbridge, Lethbridge, Alberta, Canada; c Department of Chemistry and Biochemistry, University of Lethbridge, Lethbridge, Alberta, Canada; d Department of Clinical Neurosciences, Cumming School of Medicine, University of Calgary, Calgary, Alberta, Canada; e Hotchkiss Brain Institute, University of Calgary, Calgary, Alberta, Canada; f Division of Physical Medicine and Rehabilitation, University of Alberta, Edmonton, Alberta, Canada

**Keywords:** Metabolomics, ^1^H NMR spectroscopy, Urine, Spinal cord injury, Biomarkers, Neurorehabilitation, Functional recovery

## Abstract

Current assessments of recovery following spinal cord injury (SCI) focus on clinical outcome measures. These assessments bear an inherent risk of bias, emphasizing the need for more reliable prognostic biomarkers to measure SCI severity. This study evaluated fluid biomarkers as an objective tool to aid with prognosticating outcomes following SCI. Using a ^1^H nuclear magnetic resonance (NMR)-based quantitative metabolomics approach of urine samples, the objectives were to determine (a) if alterations in metabolic profiles reflect the extent of recovery of individual SCI patients, (b) whether changes in urine metabolites correlate to patient outcomes, and (c) whether biological pathway analysis reflects mechanisms of neural damage and repair. An inception cohort exploratory pilot study collected morning urine samples from male SCI patients (n=6) following injury and again at 6-months post-injury. A 700 MHz Bruker Avance III HD NMR spectrometer was used to acquire the metabolic signatures of urine samples, which were used to derive metabolic pathways. Multivariate statistical analyses were used to identify changes in metabolic signatures, which were correlated to clinical outcomes in the Spinal Cord Independence Measure (SCIM). Among SCI-induced metabolic changes, biomarkers which significantly correlated to patient SCIM scores included caffeine (R = -0.76, p < 0.01), 3-hydroxymandelic acid (R= -0.85, p < 0.001), L-valine (R = 0.90, p < 0.001; R = -0.64, p < 0.05), and N-methylhydantoin (R = -0.90, p < 0.001). The most affected pathway was purine metabolism. These findings indicate that urinary metabolites reflect SCI lesion severity and recovery and provide potentially prognostic biomarkers of SCI outcome in precision medicine approaches.

## Highlights

• ^1^H nuclear magnetic resonance (NMR) spectroscopy identified robust urine biomarkers in patients with spinal cord injury (SCI).
• Quantitative NMR-based metabolomics identified metabolic profiles linked to the extent of recovery among SCI patients.
• Changes in urine metabolites correlated with patient functional outcomes.
• Metabolomic biomarker analysis addresses the need for reliable prognostic biomarkers of spinal cord injury.

## 1 Introduction

Spinal cord injuries (SCIs) can have long-term consequences for survivors and their families. In Canada, there are approximately 4300 cases annually (Noonan et al., 2012), generating a significant economic burden to society. Rehabilitation represents the primary approach to promote long-term functional recovery after SCI, which can occur due to compression, incision, or contusion (Nas et al., 2015). Nevertheless, there is an urgent need to promote evidence-based rehabilitation therapies to optimize the potential for recovery. Currently, the American Spinal Cord Injury Association (ASIA) Impairment Scale serves as a measure of prognostic outcomes following SCI (Spiess et al., 2009). Additionally, computerized tomography and magnetic resonance imaging remain important imaging modalities for diagnosing and determining SCI severity (Metz et al., 2000); however, the large expenses and low throughput are major pitfalls associated with these technologies (Goeree et al., 2005, Krueger et al., 2013, Tator et al., 2016). Currently, there is no single “gold standard” prognostic biofluid marker to objectively determine if a patient has suffered an SCI and if so, the extent of physical and functional disability. This results in the demand for a high-throughput method that can rapidly diagnose SCI severity and optimize the potential for subsequent recovery.

The present study utilized metabolomics as a powerful approach to provide quantitative assessment of endogenous small molecules within biological fluids, such as urine (Nicholson and Lindon, 2008). NMR has the most number of detectable (209) and unique (180) metabolites in human urine when compared to chromatography and mass spectrometry techniques (Bouatra et al., 2013). Previous work from our laboratories has demonstrated that levels of metabolic compounds in bio-fluids can be used to effectively predict recovery following brain injury (λ) and stress due to a natural disaster (Paxman et al., 2018). Similar changes in metabolism may also accompany SCI. Metabolic changes are accompanied by severe atrophy of denervated musculature, which leads to marked changes in body composition (Baumann and Spungen, 2000, Gorgey et al., 2013). Metabolic rates decline due to the loss of metabolically active lean body mass below the level of the lesion and a corresponding increase in adiposity (Giangregorio and McCartney, 2016). Accordingly, SCI diminishes whole body glucose transport, which is proportional to the reduction in muscle mass (Spungen et al., 2003) and can provoke disorders in carbohydrate and lipid metabolism (Baumann and Spungen, 1994). Following cervical SCI, disrupted glucose homeostasis may downregulate gene expression related to lipid oxidation and glycogen storage in skeletal muscle (Long et al., 2011). SCI is also accompanied by insidious delayed secondary tissue damage that can persist for months of years (Popovich et al., 2002, Donnelly and Popovich, 2007, Allan and Rothwell, 2003, Schwab and Bartholdi, 1996). Thus, knowledge of the biochemical pathways altered by SCI will inform about the natural changes following initial injury throughout the recovery process. A robust prognostic metabolic biomarker that reflects these changes would be valuable to improving SCI treatment and patient outcome.

Here, we identified a metabolic fingerprint in urine of SCI patients within 1 month of injury and at 6 months post-injury using nuclear magnetic resonance (NMR) spectroscopy. Using both univariate statistics and a machine learning multivariate approach, the present study determined (1) the metabolomics profile of SCI patients initially and long-term after injury; (2) which metabolites lead to the observed differences; (3) which biochemical pathways contribute to these metabolomic alterations; (4) the accuracy of the identified metabolites as diagnostic SCI biomarkers; and (5) the prognostic value of the biomarker profiles in predicting personal clinical outcome.

## 2 Results

### 2.1 Metabolic biomarkers related to functional improvement

**Fig. 1:**
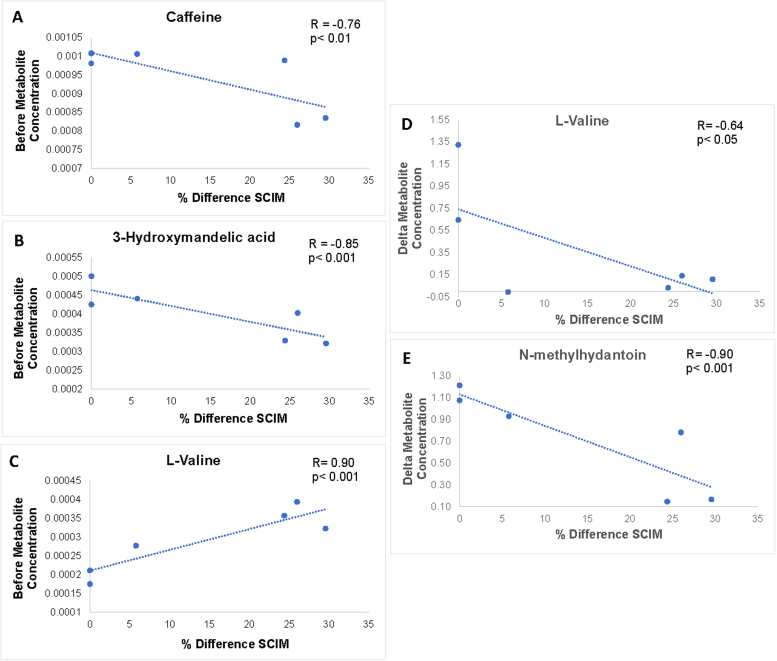
Pearson R correlations showing the correlation between both initial metabolite concentration (A-C) and delta in the metabolite concentration (D, E) to the percentage difference in the patient SCIM scores. Improved patient recovery corresponds to a higher percentage difference in the SCIM score. The R and *P-*values for each correlation are provided in the top right of each figure.

Clinical improvement was evident amongst the SCI patients at 6-months post-injury compared to the initial scores (1 month post-injury) for the SCIM. The average improvement was 10.8 ± 10.4 points. To determine if initial metabolite concentrations can predict this functional improvement, Pearson R correlations, were computed between initial metabolite levels and % difference in SCIM scores, revealing three urinary metabolites with significant correlations: caffeine (R = -0.76, *p* < 0.01; Fig. 1A), 3-hydroxymandelic acid (R = -0.85, *p* < 0.001; Fig. 1B), and L-valine (R = 0.90, *p* < 0.001; Fig. 1C). To determine if the change in metabolite concentration served as a proxy measure of the degree of recovery, Pearson R correlations were also computed between the difference in metabolite concentrations and % difference in SCIM scores, revealing significant correlations for L-valine (R= -0.64, *p* < 0.05; Fig. 1D) and N-methylhydantoin (R= -0.90, *p* < 0.001; Fig. 1E).

### 2.2 Metabolomic profiles are robust predictors of recovery following SCI

**Fig. 2:**
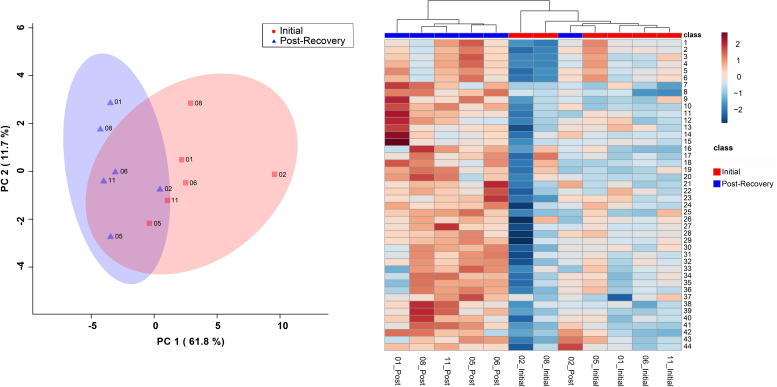
Principal Components Analysis (PCA) scores plot (left) and heat map (right) representing unsupervised separation and hierarchical clustering analysis of male SCI patients’ metabolic profiles. The legend indicates the class label: initially one week after SCI and 6 months post-injury. The heat maps illustrate up-regulation versus down-regulation of metabolites significant by the VIAVC best subset (n = 3 bins) and paired *T*-test/Wilcoxon Mann–Whitney Test (n = 44 bins). Supplementary Table 1 provides the name of the metabolite corresponding to each of the numbers provided to the right of the heat map.

**Fig. 3:**
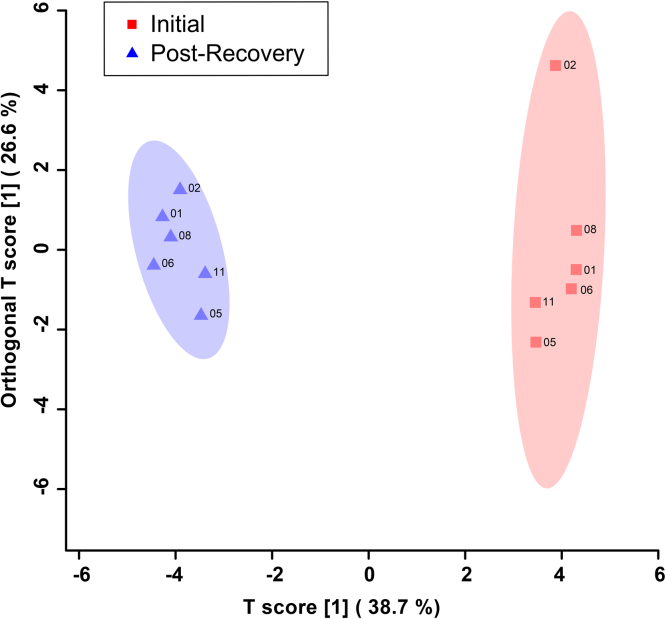
Orthogonal Projections to Latent Structures Discriminant Analysis (OPLS-DA) score plot showing supervised separation between male SCI patients initially (red/squares) and 6 months post-injury (indigo/triangles). This analysis was carried out using a list of urinary metabolites found to be statistically significant by either the paired *T*-test/Mann–Whitney or VIAVC testing. The 95% confidence interval is indicated by the shaded ellipses. The x- and y-axis show the predictive (between group) and orthogonal (within group) variation, respectively. The following are the cross-validation and permutation measures for the OPLS-DA figures: R^2^Y = 0.991, *p* = 0.011; Q^2^ = 0.808, *p* = 0.002.

The bins found to be significant by either the paired *T*-test/Wilcoxon Mann–Whitney test (n = 44 bins) or the VIAVC best subset (n = 3 bins) were used for subsequent analysis. The VIAVC best subset consisted of dopamine, Sumiki’s acid, and caffeine. PCA and heat map illustration demonstrated a partial degree of unsupervised group separation (Fig. 2A and B). The OPLS-DA plot (Fig. 3) illustrates significant group separation at 1 month and 6 months (R^2^Y = 0.991, *p* < 0.05; Q^2^ = 0.808, *p* < 0.01). This supervised model indicated a change in the metabolic profiles over the course of patient recovery in repeated samples. Metabolites that contributed the most to the group separation are shown in Appendix 1, ranked in order of significance according to the paired *T*-test/Wilcoxon Mann–Whitney test.

**Fig. 4:**
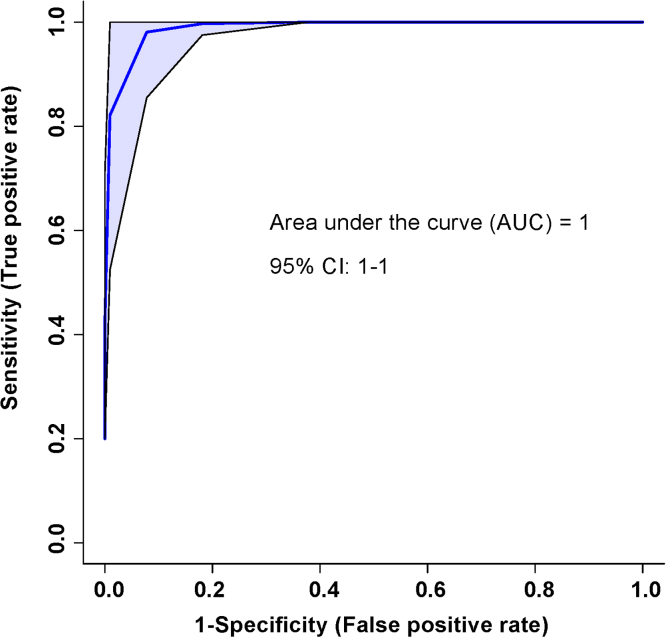
The Receiver Operator Characteristic (ROC) curve represents a high sensitivity and specificity of the group separation between initial and post-injury samples. The corresponding area under the curve (AUC) and confidence interval are indicated on each figure. The ROC curve was constructed using the metabolites determined to be significantly altered based on the VIAVC best subset, which corresponds to 3 bins.

A ROC curve was also generated for the SCI patients. An area-under-the-curve equal to 1 was achieved, with a 95% confidence interval of 1-1 (Fig. 4). The predictive accuracy for this curve when 2 bins were included was 92% and when all 3 bins were included was 100%. This analysis was based on the 3 bins significant by the VIAVC best-subset.

**Fig. 5:**
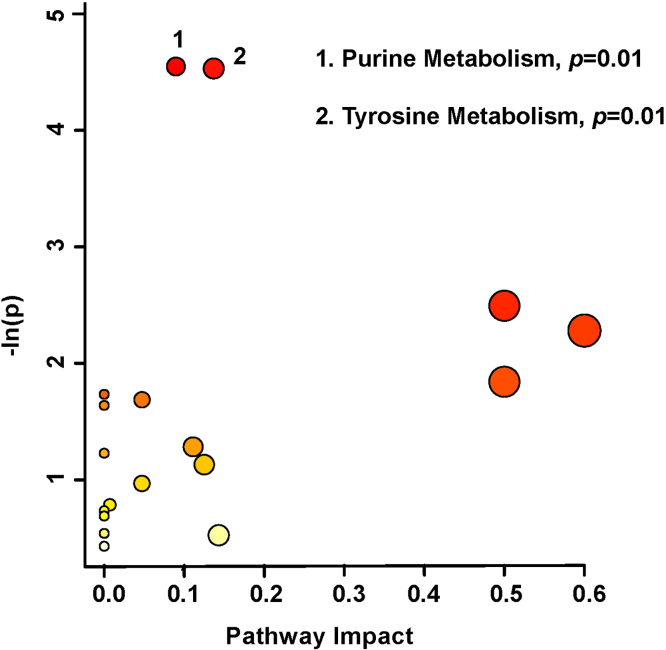
Metabolic pathway analysis, conducted based on spectral bins that are significant by either the VIAVC best subset or the paired *T*-test/Wilcoxon Mann-Whitney test. A higher value on the y-axis indicates a lower *p*-value for the pathway and the x-axis provides the pathway impact, which is a measure of how affected each pathway is by the metabolites identified as significantly altered. The color of each circle is an indication of the p-value, with darker colors being more significant. The size of the circle is proportional to the pathways impact factor. Only pathways with a *p*-value less than 0.05 are labeled. (For interpretation of the references to color in this figure legend, the reader is referred to the web version of this article.)

Pathway topology analysis (Fig. 5) illustrated the impact of urinary metabolites on changes to the SCI patients’ metabolic profiles, presented in increasing order of impact. Metabolic pathways significantly affected amongst the patients were purine metabolism (*p* < 0.01), followed by tyrosine metabolism (*p* < 0.01). Pathway analysis was based on bins significant by the VIAVC best subset, paired *T*-test, and Wilcoxon–Mann–Whitney test.

## 3 Discussion

Here we show that metabolomic profiles in urine change throughout recovery following SCI, and positively relate to SCIM scores. The most significant change occurred in metabolites that were part of the VIAVC best subset (dopamine, Sumiki’s acid, and caffeine), suggesting that these metabolites present the most robust indicators of successful recovery in SCI. Their value as metabolic biomarkers of SCI severity was confirmed by a predictive accuracy of 100%. The main metabolic pathways altered by recovery following SCI included purine metabolism and tyrosine metabolism. Moreover, we showed that the degree of functional recovery is best predicted by caffeine, 3-hydroxymandelic acid, and L-valine. Thus, a metabolomics approach combined with machine learning is able to provide clinically accessible biomarkers with prognostic potential for SCI recovery.

Purine metabolism presented as the most significantly affected pathway in SCI recovery. This follows from our previous work (Bykowski et al., 2021), which indicated that urinary purines and their derivatives may play a role in pathology arising from traumatic brain injury. Purines are known for their neuroprotective roles in the nervous system, with the ability to mitigate inflammatory responses (Jackson et al., 2016). Although it is well known that purine metabolism is disrupted in the wake of head trauma (Clark et al., 1997, Cronstein, 1994), there is less evidence for their dysregulation following SCI. However, a recent study in which hypoxia was induced in a SCI rat model led to an increase in extracellular purine derivatives, specifically adenosine and inosine (Takahashi et al., 2010). Inosine, which is implicated within the purine pathway in our study, was also shown to be present at ten-fold higher concentrations than adenosine. The excretion of this purine in the urine reflects its presence within the body as an endogenous neuroprotective agent against inflammation. Furthermore, levels of purine derivatives were also shown to vary with intracellular calcium concentration within the spinal cord, whereby increased calcium activity promoted purine release (Liu et al., 2006). Increased osteoclast activity associated with bone fracture following SCI may a play a role in calcium fluctuations which influence purine release.

An alternative mechanism for purine release is associated with the inflammatory response following SCI, which is mediated by microglia. As the main active immune defense in the central nervous system, microglia respond to sites of injury via release of ATP (a purine derivative) from the injured area (Dou et al., 2012). This mechanism may also explain dysregulation of the purine pathway in the present study. Thus, urinary metabolites implicated in purine metabolism may be indicative of their neuroprotective action or their role in initiating immune system activation.

Evidence for tyrosine metabolites in the urine suggests low cellular uptake and excessive excretion of catecholamines produced along this pathway, including dopamine which was part of the VIAVC best subset. Although there have been few studies relating the role of dopamine to spinal cord functioning in humans, evidence in model systems such as the rodents, lamprey and *C. elegans* showed sources of dopamine in the spinal cord which modulate movement patterns (Sharples et al., 2014). Although this area of study is in its infancy, extrapolation of this finding to humans is plausible. The loss of dopaminergic as well as noradrenergic inputs to the spinal cord following SCI may contribute to the loss of rhythmic and autonomic movements(Han et al., 2007; Acton et al., 2018). Additionally, it was shown that sources of dopamine in the dorsal horn of the rat spinal cord modulate the bladder reflex, and underlies micturition following SCI (Hou et al., 2016). Thus, spinally derived dopamine in animal models increases the possibility of a similar mechanism in human patients.

Caffeine presented as a clinically significant metabolite, with a negative correlation to patient SCIM scores. The role of caffeine in injury to the central nervous system is still not fully understood and is subject to debate. A recent review indicated that caffeine may be utilized as an adenosine receptor antagonist and have therapeutic, neuroprotective, and pain relief roles (Rivera-Olive and Diaz-Rios, 2014). However, a more recent study of spinal cord injury in a rat model has shown that caffeine may have a neurotoxic role associated with reduced neural repair and increase inflammation (Yang and Jou, 2016). Changes in caffeine levels may also be related to patient mobility and metabolic rates. For example, SCI patients experience a reduced metabolic rate as a result of skeletal muscle atrophy and lower levels of activity (Graham-Paulson et al., 2017). Delayed caffeine absorption was found to be greater for tetraplegic than for paraplegic SCI patients, owing to a greater loss of skeletal muscle and subsequent reduction in resting metabolic rate (Graham-Paulson et al., 2017). In tetraplegia, gastrointestinal emptying times are prolonged, with effects experienced as soon as three days after sustaining an SCI injury (Qualls-creekmore et al., 2009). Therefore, caffeine likely serves as a biomarker of metabolic slowing, and a higher risk of adiposity associated with a reduced decreased metabolic rate due to the loss of metabolically active lean body mass. Clinical intervention requires ways to boost the metabolic state of patients to prevent this downstream effect. With rehabilitation and an increase in patient mobility, increase muscle strength may out-weigh atrophy, and with improved metabolism and absorption of nutrients, the ensuing decrease in urinary caffeine levels may indicate recovery. Further studies of caffeine in human cohorts of SCI patients should focus on metabolomic changes in both urine and blood, as this would provide a more wholistic view of how caffeine is being utilized and processed in the body. It is also important to note that dietary intake is the only source of caffeine in the metabolome, as it is not endogenous to the human body. Although this study made use of a paired univariate test, it did not account for patient diet. Future studies into the role of urinary caffeine levels as a biomarker for SCI recovery should control for dietary intake of caffeine to eliminate this possible confounding factor.

In correlation with functional recovery, 3-hydroxymandelic acid revealed a negative relationship to improved recovery, as its levels decreased. In a previous study, it was found that elevated excretion of 3-hydroxymandelic acid is associated with tyrosine intake (Fell et al., 1979). Excretion of this catecholamine metabolite is additional evidence supporting dysregulation of the tyrosine metabolic pathway as indicated in the pathway analysis for this study. This suggests a corresponding decrease in excretion of tyrosine derivatives as recovery progresses, as inferred from the negative correlation.

Lastly, L-valine, an essential amino acid, is a building block for muscle tissue, presented with a positive correlation to % difference measurements at the SCIM. Muscle atrophy is a cardinal feature of SCI (Giangregorio and McCartney, 2016); it is likely that excretion of L-valine in the urine is indicative of muscle breakdown following SCI, and overall liberation of its constituent amino acids. The fact that an initial increase in its levels is associated with recovery likely underlies the body’s subsequent demands for protein building blocks to restore muscle tissue.

This study also aimed to determine if the change in metabolite concentration could serve as a proxy measure of the degree of recovery. L-valine again presented as a significant metabolite for this comparison, but with a negative correlation. Neuronal death following SCI is triggered by an elevation in intracellular calcium levels (Tymianski et al., 1993), and L-valine is postulated to be a part of this pathway (Simpson et al., 1990). As an excitatory, branched chain amino acid implicated in the cascade that leads to calcium-induced neuronal cell death, it follows that a decrease in its levels is associated with an improvement in patient recovery.

A negative correlation was also observed for N-methylhydantoin levels compared to the % difference SCIM scores. N-methylhydantoin is a by-product of the degradation of creatinine by bacteria (Seifter, 2014). Creatinine is a breakdown product of creatine phosphate in the muscle, and therefore indicates muscle atrophy. Unlike most polar substances, creatinine is not reabsorbed by the kidneys, and is filtered out to be excreted in the urine (Levey et al., 1998). Decreased levels of N-methylhydantoin post-injury may indicate a decrease in skeletal muscle atrophy, via reduced creatinine levels.

Although the sample size of the present study was limited, the pairing of spinal cord injured subjects within this pilot study ensures that the regulation of metabolite concentrations is significant across the initial and post-injury groups. Identification of unique urinary metabolic signatures is validated via this paired analysis, which increases statistical power of this pilot study. Another limitation to this pilot study is that patients’ diets were not controlled while in hospital or following release. Lastly, the effects of body mass index, drug treatments, and medical history were not controlled for in this study due to the small size of the available patient cohort. A recent study of human serum samples has shown that diet, along with several other lifestyle factors, can be predictive of 76% of the metabolome (Bar et al., 2020); however, this study utilized liquid chromatography-mass spectrometry, instead of NMR, and did not investigate urine. In the present study, the effects of these confounding factors are minimized by the fact that two urine samples were collected from each patient allowing for a paired univariate analysis; thus, significant changes to metabolite levels reflect a global change across all *paired* urine samples.

The identified biomarkers and metabolic pathways may represent attractive therapeutic targets and have potential for clinical translation. Metabolites with statistically significant correlations to SCIM outcomes represent a window of opportunity for neurotherapeutic intervention amongst spinal cord injured patients. Caffeine, 3-hydroxymandelic acid, and L-valine may have the ability to predict recovery outcomes, whereas N-methylhydantoin and L-valine have potential to serve as measures of the change in metabolic profiles over time.

## 4 Experimental procedure

### 4.1 Patient characteristics and sample collection

This study was embedded in a larger study entitled the UCAN study, which follows patients with SCI, stroke, and traumatic brain injury throughout their recovery from one month to 6 months post-injury. Male patients with incomplete (n = 4) and complete SCI (n = 2) were recruited through the Foothills Medical Centre, University of Calgary (average age 55 ± 20 years; Table 1). Pairs of fasting morning urine samples (acquired between 6 a.m. and 9 a.m.) were collected at two different time points: one month following injury and again at 6 months post-injury. Pairing the samples for this within-subject study reduced the influence of confounding factors, such as diet, lifestyle, body mass index, medical history, and drug treatments, to raise the validity of the analysis. Urine samples were stored at -80 ℃ until further processing.

**Table 1:** Patient characteristics table indicating the age, lesion location, co-morbidities, and SCI type, as well as both the initial and post-injury SCIM scores.

Patient Code	SCI Type	ASIA Score	Lesion Location	Co-Morbidities	Age	Pre SCIM	Post SCIM
**SCI_01**	Incomplete	D	Central Cord		80	84	89
**SCI_02**	Complete	A	T7		29	70	70
**SCI_05**	Incomplete	D	C4		38	72	92
**SCI_06**	Complete	A	T6		50	49	66
**SCI_08**	Incomplete	D	C6-C7		59	100	100
**SCI_11**	Incomplete	B	C2-C4	UTI, C2-C3 spinal artery infarct	73	77	100

We initially received seven pairs of urine samples, of which one was a female. To remove the effect of sex as a confounding factor, we removed this female from the multivariate/univariate statistical analysis presented in this pilot study. The present research was approved by the University of Calgary Conjoint Health Research Ethics Board (CHREB) and the University of Lethbridge Human Participant Research Committee in accordance to the standards set forth by the Tri-Council Policy Statement: Ethical Conduct for Research Involving Humans.

### 4.2 Clinical assessments

The Spinal Cord Independence Measure (SCIM) was completed for each patient at 1 month following injury and at 6 months follow-up. The SCIM, based on patient self-reports, includes the following areas of function: self-care (sub-score 0–20), respiration and sphincter management (0–40), and mobility (0–40) (Catz et al., 1997). The final score ranges from 0 (total dependence) to 100 (total independence). We also collected information on SCI type (complete or incomplete), ASIA score, sex, lesion location, co-morbidities, and age (see Table 1).

### 4.3 NMR sample preparation, data acquisition, and processing

To control for pH and reduce positional noise within NMR-generated datasets (Gil et al., 2016, Smelter et al., 2017), urine samples were combined with buffer consisting of 4:1 ratio of dibasic potassium phosphate (K_2_HPO_4_) to monobasic potassium phosphate (KH_2_PO_4_) with a combined concentration of 0.625 M in dH_2_O (pH 7.4), containing 3.75 mM NaN_3_ anti-microbial agent and 0.375 M potassium fluoride (KF). For sample preparation, 400 µL of urine, 160 µL of buffer, and 40 µL of 0.02709% weight/volume D_2_O with trimethylsilyl propanoic acid (TSP) were pipetted into a microfuge tube. Each sample was centrifuged at 12,000 rpm for 5 min at 4 ℃ to eliminate insoluble matter. 550 µL of supernatant was then transferred to an NMR tube, vortexed and loaded into the spectrometer. All final NMR samples were pH checked to 7.4 ± 0.05 with an NMR pH meter.

A 700 MHz Bruker Avance III HD NMR spectrometer and a room-temperature TBO probe were used to acquire the NMR data. Three-dimensional and one-dimensional shimming experiments were conducted prior to NMR data acquisition to correct for any inhomogeneities in the static magnetic field. The data were acquired using a one-dimensional 1H Nuclear Overhauser Effect Spectroscopy experiment with water suppression, 128k points, and 128 scans. The data were processed using zero filling to 256k points, line broadening to 0.3 Hz, and automatic phase and baseline correction. The spectra were then imported into MATLAB where they underwent dynamic adaptive binning (Anderson et al., 2011), followed by manual inspection and correction of the bins, and recursive segment-wise peak alignment (Veselkov et al., 2009). In total, 505 bins were created for this analysis.

Metabolites were identified using a combination of resources: Chenomx 8.2 NMR Suite (Chenomx Inc., Edmonton, Alberta, Canada), the Human Metabolome Database (HMBD) (Wishart et al., 2018), and the Human Urine Metabolome (Bouatra et al., 2013) containing a list of NMR-derived urinary metabolites and their concentrations.

### 4.4 Statistical analysis

Metabolic pathway and multivariate testing and visualization was carried out using MetaboanalystR version 2.0.4 package running inside R version 3.5.3 (Pang et al., 2020). Pathway topology analysis was conducted by selecting the hypergeometric test for the over-representation analysis, relative-betweenness centrality for the topology analysis and using the Kyoto Encyclopedia of Genes and Genomes (KEGG) database for Homo sapiens as the pathway library (Wishart et al., 2018, Xia and Wishart, 2010). Only the metabolites identified as significantly altered in this study (Supplementary table 1) were listed when carrying out pathway topology analysis. Multivariate statistical analysis was used to determine if urinary metabolite profiles could be used to distinguish between the 1 month and 6 month-post-injury samples. Prior to modeling, the data were normalized to the total metabolome (excluding the regions corresponding to water and urea), log transformed, and pareto-scaled (Craig et al., 2006, Wiklund et al., 2008, van den Berg et al., 2006, Box and Cox, 1964). Bins containing significant metabolites were sorted according to the F-ranked Variable Importance Analysis based on random Variable Combination (VIAVC) analysis (Yun et al., 2015) to identify significant metabolites based on the Receiver Operator Characteristic (ROC) and the subsequent Area-Under-the-Curve (AUC) analysis (Fawcett, 2005). It also employs a binary matrix resampling method as a robust method for random data sampling data, and all multivariate supervised models underwent double ten-fold cross-validation (DCV) and permutation testing using 2000 permutations (Szymanska et al., 2012). The DCV algorithm utilized by VIAVC sets aside an independent test set that is used to validate the model. This process of model validation is repeated multiple times, with a different randomly selected test set used each time, until every sample has been included in the test set at least once. Univariate statistical tests were also conducted; either a paired *T*-test or paired Wilcoxon-Mann-Whitney test was used in the case of parametric or non-parametric data, respectively. A Shapiro-Wilk test was used to determine if the data for each bin was parametric or not (Goodpaster et al., 2010).

An orthogonal projection to latent structures discriminant analysis (OPLS-DA) was conducted to visualize between-group separation as a function of within-group separation (Wiklund et al., 2008). This was complemented by a Principal Components Analysis (PCA), which demonstrated the degree of separation between the groups without the presence of an algorithm, as well as unsupervised hierarchical clustering illustrated by the accompanying heat map.

Pearson R correlations were computed between the percent difference of the patients’ SCIM scores and both the initial and delta of the normalized urinary metabolite concentrations (Fig. 1). Significance was based on the Bonferroni corrected *p*-value, obtained by dividing alpha< 0.05 by the number of VIAVC F-ranked bins tested for this analysis (n = 19), to obtain a rigorous set of clinically relevant metabolites (Goodpaster et al., 2010). The delta for the urinary metabolites was calculated by subtracting the post-injury normalized concentrations from the initial normalized concentrations. The % difference for SCIM scores at the two different time points were computed as follows, based on the clinical data provided in Table 1:

$$\left(\frac{\mathrm{Post}\mathrm{Recovery}\mathrm{Score}-\mathrm{Initial}\mathrm{Score}}{\left(\frac{\mathrm{Post}\mathrm{Recovery}\mathrm{Score}+\mathrm{Inital}\mathrm{Score}}{2}\right)}\right)*100\%$$

## Ethical Information

The present research was approved by the University of Calgary Conjoint Health Research Ethics Board (CHREB) and the University of Lethbridge Human Participant Research Committee in accordance to the standards set forth by the Tri-Council Policy Statement: Ethical Conduct for Research Involving Humans.

## CRediT authorship contribution statement

S.D., C.O. and C.D. did overall study design and G.M. and T.M. developed and designed the metabolomic portion of the study. C.H. and C.D. recruited the participants. S.D. collected the primary clinical data. E.B. and J.P. prepared the urine samples for NMR data acquisition and performed spectral analysis. E.B., J.P., C.D., T.M. and G.M. contributed to writing the main manuscript. All authors have approved the final version of this manuscript.
