# Supplementary material for: Urinary biomarkers indicative of recovery from spinal cord injury: A pilot study
Source: IBRO Neurosci Rep. 2021 Feb 18;10:178–85. doi: 10.1016/j.ibneur.2021.02.007 (PMC8020035; doi:10.1016/j.ibneur.2021.02.007)
Supplement: Supplementary file 1 — Supplementary material. [file mmc1.docx]

**Supplementary Table 1.** Statistically significant urinary metabolites amongst a male population of spinal cord injured patients, according to the paired T-test/Wilcoxon Mann-Whitney tests and VIAVC F-ranked analysis. Metabolites are ranked in order of significance (*p*<0.05) according to the paired T-test/Wilcoxon Mann-Whitney test; those with associated *p*-values for the VIAVC F-ranked test are also reported. Heat map numbers indicate metabolites that correspond to labelling on the heat map (Figure 2). Single dagger indicates the metabolite is part of the VIAVC F-ranked set and the double dagger indicates the metabolite is part of both the VIAVC best-subset and F-ranked.

| **Heat Map Number** | **Metabolite** | **Chemical Shift (ppm)** | **Paired T/Wilcoxon *p* value** | **Regulation** |
| --- | --- | --- | --- | --- |
| 4 | 4-Pyridoxic Acid | 2.348 | 0.0011 | Up |
| 19 | Nicotinurate | 8.773 | 0.0015 | Up |
| 41 | Sumiki’s acid †† | 6.494 | 0.0029 | Up |
| 25 | Methylimidazoleacetic Acid | 7.036 | 0.0036 | Up |
| 3 | Imidazole | 7.252 | 0.0038 | Up |
| 7 | Gluconic acid † | 4.116 | 0.0059 | Up |
| 32 | Adenosine Monophosphate | 6.148 | 0.0065 | Up |
| 38 | 4-Hydroxy-3-Methylbenzoic Acid | 7.699 | 0.0071 | Up |
| 42 | N-Methylhydantoin † | 2.929 | 0.0078 | Up |
| 20 | Xanthosine | 4.402 | 0.0081 | Up |
| 34 | 3,4-Dihydroxybenzeneacetate | 6.740 | 0.0089 | Up |
| 18 | Indoxyl Sulfate | 7.732 | 0.0113 | Up |
| 17 | Guanosine | 4.424 | 0.0129 | Up |
| 36 | Isoferulic Acid | 6.393 | 0.0141 | Up |
| 39 | 2-Furoic Acid | 6.563 | 0.0149 | Up |
| 40 | 3-Methylhistamine † | 8.034 | 0.0163 | Up |
| 11 | Hydroxycobalamin | 6.613 | 0.0164 | Up |
| 16 | Formate | 8.461 | 0.0195 | Up |
| 5 | Acetoacetate | 2.288 | 0.0215 | Up |
| 21 | Dopamine †† | 6.862 | 0.0216 | Up |
| 1 | Glutarylglycine | 2.134 | 0.0218 | Up |
| 9 | N-Acetyltyrosine † | 7.769 | 0.0269 | Up |
| 12 | L-Trytophan | 3.502 | 0.0275 | Up |
| 35 | Kynurenic Acid | 6.706 | 0.0277 | Up |
| 22 | D-Glucuronic Acid | 4.111 | 0.0284 | Up |
| 33 | Inosine | 6.125 | 0.0295 | Up |
| 37 | Imidazole † | 8.146 | 0.0295 | Up |
| 26 | 2-Furoylglycine | 6.563 | 0.0313 (W) | Up |
| 31 | ADP | 6.156 | 0.0313 (W) | Up |
| 29 | Pyroglutamic Acid | 4.172 | 0.0313 (W) | Up |
| 13 | Fructose | 4.104 | 0.0313 (W) | Up |
| 27 | Riboflavin | 2.580 | 0.0313 (W) | Up |
| 10 | Indole-3-lactate † | 7.757 | 0.0313 (W) | Up |
| 14 | Imidazole † | 8.146 | 0.0313 (W) | Up |
| 15 | 2-Isopropylmalic acid † | 2.691 | 0.0313 (W) | Up |
| 8 | Caffeine †† | 3.368 | 0.0313 (W) | Up |
| 24 | 2,2-Dimethylsuccinic Acid | 2.700 | 0.0318 | Up |
| 30 | 6-Hydroxynicotinate | 6.625 | 0.0371 | Up |
| 6 | Succinylacetone | 2.276 | 0.038 | Up |
| 28 | Pyroglutamic Acid | 4.177 | 0.0421 | Up |
| 23 | 4-Methylcatechol | 6.797 | 0.0435 | Up |
| 44 | Deaminotyrosine † | 6.875 | 0.0474 | Up |
| 43 | Homovanillate | 6.887 | 0.0484 | Up |
| 2 | N-Acetylmannosamine | 2.124 | 0.0488 | Up |

† indicates significant by VIAVC F-ranked

†† indicates significant by VIAVC F-ranked and VIAVC best subset
